# Injection practices in Nepal: health policymakers’ perceptions

**DOI:** 10.1186/1472-698X-14-21

**Published:** 2014-06-24

**Authors:** Sudesh Gyawali, Devendra Singh Rathore, Pathiyil Ravi Shankar, Manisha Maskey, Vikash Kumar KC

**Affiliations:** 1 Department of Pharmacology, Manipal College of Medical Sciences, Pokhara, Nepal; 2 Department of Pharmacy, L.R. Institute of Pharmacy, Jabli-kyar, Solan, India; 3 Department of Pharmacology, Xavier University School of Medicine, Oranjestad, Aruba, Kingdom of the Netherlands; 4 Department of Community Medicine, Manipal College of Medical Sciences, Pokhara, Nepal; 5 Department of Statistics, PN Multiple Campus, Pokhara, Nepal

**Keywords:** Drug policy, Hazardous waste, Health policy, Injection, Needle stick injury, Nepal

## Abstract

**Background:**

The unnecessary and unsafe use of injections is common in developing countries like Nepal. Policymakers have an important role in promoting rational and safe injection use. Hence, the present study was carried out to explore the perception of health policymakers regarding safe injection practice in Nepal.

**Methods:**

An exploratory qualitative study design was used in this study. Key policymakers from both the central and regional level were selected using purposive sampling. A semi-structured questionnaire advocated by the World Health Organization (WHO) was used after modifying the context. Interviews were conducted to clarify doubts and obtain additional information. The data was analyzed manually using deductive content analysis technique.

**Results:**

In total, eleven policymakers participated. All unanimously agreed that injection safety is a problem and seven participants reported that injections are overused. They shared the opinion that injections are administered by various providers, including formal and informal health providers, and also quacks. Almost half the respondents reported that the National Drug Policy discourages injection overuse, while others reported that the policy contains no provisions regarding injection overuse. Most policymakers stated that only single-use disposable injection equipment is used to provide injection, while others thought that sterilizable glass syringe is also used. More than half of the participants believed that the quality of injection equipment available in the Nepalese market is not regulated by any government institution. Almost two-third of the policymakers stated that syringes and needles are not reused, while the rest thought syringes might be reused without sterilization in some parts of the country. Almost half of the respondents stated that illegal commercialization of used syringes exists in Nepal. Almost all respondents thought that health care institutions have a waste management plan, while more than half of them opined that such plans are limited to tertiary care hospitals located in the capital.

**Conclusions:**

The result of this study revealed a divergence of views among policymakers, even among those in the same ministry. Though there has been some effort from the government to increase the safety of injection practices, greater efforts are required, especially with regard to standardization of policies and procedures related to injection practice.

## Background

### Country overview

Nepal is a small landlocked country in South Asia, sandwiched between the two giant countries of India and China with a population of 26.5 million [1]. It ranks as one of the poorest countries in the world, with a per capita income of US $717 [1]. For administrative purposes, Nepal is divided into 5 development regions, 14 zones and 75 districts [1]. The government of Nepal administers health care services through the Ministry of Health and Population (MoHP). MoHP delivers preventive, promotional and curative health services through the Department of Health Services (DoHS). In each development region of Nepal, there is a regional health directorate and in each district, there is a public health office. In Nepal, the government provides health care services through outreach clinics, female community health volunteers, primary health care facilities [Sub Health Posts (SHP), Health Posts (HP), Primary Health Care Centers (PHCC)] and other hospitals (district, zonal, regional and central). Apart from this, the ministry also controls, monitors and supervises health care services provided by the non-governmental sector [2,3]. The regional health directorates and district public health offices, under the supervision of the MoHP, have the responsibility of monitoring, controlling and ensuring the delivery of health services to people under their jurisdiction [2].

Although Nepal is divided into different administrative units, most of the administrative power is centralized. Hence, most important policies and plans for the country are implemented and regulated centrally from the Ministry level [4]. Following a decade long violent conflict in the country that lasted almost two decades, currently the country is passing through political transition and a debate on state restructuring and administrative reform is ongoing in the Constituent Assembly [5].

### Injection use

Injection is an important health care procedure. People receive (use) injections from the beginning of life as a vaccine (immunization purpose) and may use them till the last day of life (curative purpose). Every year billions of injections are used worldwide and most (>95%) are used for therapeutic purposes [6]. Even though injection use is a global phenomenon, its overuse in developing countries is very common [7]. There are various factors that make injections popular in developing countries. Some of these factors are [8-10]:

1. Lack of adequate knowledge of prescribers about rational use of injections

2. Demand for injections by patients because they perceive injections to be more powerful than oral formulations

3. Economic benefits to the injection providers

4. Weak policy and laws regulating injection practice.

A survey conducted to estimate worldwide injection use frequency (per person per year) showed that injection use in the Southeast Asia region D (which includes Nepal) was high and most injections used were neither necessary nor safe [11]. A large number of injections in the region were provided in an unsafe manner by unqualified personnel [7] reusing injection equipment without proper sterilization [11]. The reuse of injection equipment without sterilization was as high as 75% (ranging from 60%-88%) in the Southeast Asia region D [11].

### Safe and unsafe injection practices

A safe injection “does no harm to the recipient, does not expose the health worker to any risk and does not result in waste that is dangerous for the community” [6]. Sterile injection practice by qualified personnel ensures the safety of the injection recipient. Proper sharp waste collection and effective protection (including vaccination against hepatitis B) ensures the safety of the provider. Appropriate sharp waste disposal ensures the safety of the community [6].

Safe and rational use of injections could save lives, but the same injections used unsafely and excessively may also threaten life by transmitting diseases and causing complications. The transmission of Hepatitis B virus (HBV), Hepatitis C virus (HCV), and Human Immunodeficiency virus (HIV) infections through unsafe injections is of particular concern [10]. It was estimated by the WHO that in the year 2008, unsafe medical injections caused 15 million, 1 million and 0.34 million cases of HBV, HCV and HIV infections, respectively [12]. Unsafe disposal of injection equipment not only increases the risk of scavenging and commercialization (resale) of the used equipment, but also may lead to needle-stick injuries (NSIs) among health care workers and waste handlers. NSIs may transmit blood-borne infections including HIV, HBV and HCV [10]. In addition to the morbidity and mortality associated with blood-borne infections, unsafe injection practices exert a heavy indirect economic burden on society which may not be properly accounted for. Hence, it is imperative to ensure safe injection practice in poor developing countries like Nepal.

There is a paucity of studies on injection practice in Nepal. Certain studies [13,14] suggest that injection practices were unsafe, administration of unnecessary injections was common and disposal of injection equipment was poor in Nepal. A study conducted in a few government primary health care facilities [15] indicated that the Government of Nepal has taken certain initiatives to promote safe injections [3]. Use of auto-disable syringes for child immunizations, safety boxes for disposal of used syringes, recommendations for the safe disposal of filled safety-boxes, and abandoning recapping of used syringes are some of the initiatives taken for safe injection practices [3,15].

The country is in transformation and restructuring of the state and promulgation of a new constitution has been long awaited [5]. The 2007 interim constitution, has guaranteed basic health care as a fundamental right of the people of Nepal [3], which may be included in the new constitution of the country. Furthermore, the 1991 Nepal Health Policy has been recommended for revision [16]. Hence, major changes in the country’s health policies are anticipated in the coming days. The perception of health policymakers about safe injection practice has important implications for regulation and control of injection practice in the country. Hence, this study was planned to obtain information about the perception of health policymakers towards safe injection practice and the initiatives being taken by the government to promote safe injection practice in Nepal.

## Methods

An exploratory qualitative study was conducted during June and July of 2012. Key informants from the Ministry of Health and Population (MoHP), a Regional Health Directorate and a District Health office were considered for the study. A representative (head) of a NGO (non-governmental organization) advocating safe injection practices in Nepal also participated.

### Sampling method

Purposive sampling was done to select the policymakers. Eight policymakers from ministry level (various departments and divisions of MoHP), two from the regional level, one from a district health office and one NGO representative were selected. Eight policymakers from the central level were selected with regard to convenience and time limitation. Furthermore, the policymakers were the chiefs of their respective departments and the number of policymakers selected was sufficient to include all chiefs of departments or divisions of MoHP relating to injection practice. The selected policymakers ranged from the ranks of Secretary to under-secretary level in the health bureaucracy. In the health bureaucracy of Nepal, Secretary is the highest position. Under this person are the 12^th^ level officer, joint secretary, 11^th^ level officer, and under secretary (from highest to lowest) [17]. As the bureaucratic structure of Nepal is centralized and most policy matters are determined (controlled) centrally [4], more policymakers from the ministry level were chosen.

While deciding on the number of participants the concept of saturation of data which has an important role in qualitative research was also used. At the central and regional level the authors had the option of recruiting more participants if data saturation was not achieved. By the eighth interview with a central level policy maker there was a repetition of data and the new information produced minimal change to the themes and ideas already confirmed by previous interviews. The interviews with regional policymakers uncovered certain issues of importance at a regional level and confirmed the data already obtained from the central participants.

### Study method

The data was collected using the questionnaire advocated by the World Health Organization (WHO) to assess and evaluate injection practices [18] after adapting to the local context. For example, answers to questions were coded based on the terminology used in Nepal for example health care workers like health assistant (HA), community medicine auxiliary (CMA), and professionalist which are common. The sequence of the questions was modified and some questions, like “How many injections are there in the National EDL?” were deleted from the list. The instrument contains standardized and open-ended questions pertaining to injection practices (practices by injection providers and interventions related to the control/prevention of injection overuse), the reuse of injection equipment (including measures to check the re-use of syringes) and the disposal of injection-related waste.

The questionnaire was used to provide a broad framework for the interview. The face to face individual interviews were conducted in the interviewees’ office during working hours, for which prior appointments were obtained. SG conducted the interviews under the guidance of VKKC and with the help of people mentioned in the acknowledgements. PRS was involved in the review of literature and defining the different categories for coding the responses. The interviews lasted for about 40-45 minutes and the policymakers did not agree with audio recording the sessions. We could not schedule repeat interviews with the policymakers due to their busy schedule. The interviewer asked the questions as per the interview guide. But, the respondents were asked about any additional points they wanted to add regarding various topics throughout the interview. The points mentioned were noted and the draft notes were shown to the interviewees after the interview. After the transcripts were ready, they were also shared with the respondents.

### Data analysis

The data was analyzed manually by three authors (SG, MM and VKKC) using deductive content analysis techniques [19,20]. The data obtained was coded using a deductive approach where literature review and the WHO questionnaire were used to develop categories and subcategories for coding and analysis. Each respondent’s response was read several times to obtain a sense of the whole and was compared with the notes taken during the interview. Then, the contents were coded in accordance with the predetermined categories [19,20]. Additional information provided by the interviewees was placed in a new category, if required. Direct quotes were contextualized, rendered readable and presented in the habitual language of the interviewees.

### Ethical issues

The study was approved by the Nepal Health Research Council (NHRC), Kathmandu, Nepal on 17th May 2012 (Ref. No. 1233). The respondents were assured about the confidentiality of information given and written, informed consent was obtained.

## Results

Out of 12 policymakers approached, 11 agreed to take part in the study. One policymaker did not participate in the study. All the participants were male. This corresponds to the gender distribution in the higher echelons of the health bureaucracy in Nepal [21].

### Injection practice

The policymakers reported that injections are popular in Nepal. Almost all policymakers stated that they were not aware of statistics or studies that quantified the number of syringes and needles sold or used annually in the country. However, one participant quantified the volume of sale of syringes in Nepal to be 15 million units per annum. Almost two-thirds of participants shared that injections in Nepal are administered by various providers including not only formal and informal health providers but also quacks. Rest of the participants stated that injections are provided by the personnel from formal and informal health systems only and one of them stated, *“… if others* [other than formal sectors] *are practicing then that is illegal…”* (Participant number 10, P-10).

Most respondents reported that injections are overused in Nepal. The main reasons offered by them for this were:

i) Health care professionals and patients have an impression that injections are more efficacious and provide quicker relief than oral dosage forms of medicine.

ii) The profits associated with injections lure health care professionals to use them.

Almost half of the respondents reported that there is a National Drug Policy that discourages injection overuse, while others reported that there is no such provision in the drug policy of Nepal. Only three respondents said that one of the objectives of the drug Policy (1995) of Nepal is to promote the rational use of medicines in general and does not specifically concern itself with injectable medicines. Most policymakers stated that the essential drug list (EDL) and standard treatment schedule (STS), have been revised to remove unnecessary injectable medications.

### Types of syringes used

The majority of policymakers stated that only single-use, disposable injection equipment is used to provide injections, while others thought that sterilizable glass syringes are also used sometimes. Half of the participants (all from MoHP) shared that the quality of syringes and needles available in the Nepalese market is not regulated by any government institution. However, less than half the participants said that monitoring may be carried out by the Department of Drug Administration (DDA), and/or the logistics management division of the Department of Health Services (DoHS). One participant was not aware about the quality controlling authority. Only two policymakers from the central level said that there is a plan to develop guidelines for the regulation of health related products, which would cover surgical products including, but not limited to, syringes and other injectable equipment. One participant said, *“Till now, there is no Government institution to check the quality of the syringes and needles imported or locally produced in our country. But draft guidelines for regulation of Health Related Products, including needles and syringes, has been prepared”* (P-9). Similarly, another participant said that the issue of quality of syringes will be addressed after implementation of the guidelines (P-10). The NGO participant stated that the Nepal Bureau of Standards and Metrology has framed criteria for standardization of syringes, but these have not yet been implemented. A photocopy of the standardization booklet was shown to the interviewer.

### Re-use and disposal of syringes

Almost two-thirds of the participants stated that syringes and needles are not reused without sterilization, while the rest mentioned that in very remote places and/or in the Terai (flat plain) region of Nepal, syringes might be reused without sterilization. One participant mentioned, *“Yes, the syringes are reused. It has been found during our field visits that the used syringes have been collected from different hospitals and sold to local scrap dealers. In some places, local clinics have discovered the repacked, untreated syringes”* (P-8).

All respondents shared that injection equipment, diluents and safety boxes are matched (supplied in the same quantity) to the delivery of injectable drugs and vaccines to government health facilities. Almost three-quarters of respondents were of the opinion that even in the family planning programme, injection equipment is matched to deliveries of injectable contraceptives, while only two respondents said that the equipment is not matched. One of the respondents said that he did not know whether the equipment was matched with deliveries of injectable contraceptives or not. Almost two-thirds of the policymakers thought that a shortage of syringes and needles contributes to unsafe injection practices.

Most policymakers were of the opinion that HIV/AIDS prevention and care programs had communicated to the general public and health care workers (HCWs), the risk of HIV infection associated with injections. Almost half the policymakers stated that commercialization (repackaging & re-selling) of used syringes exists in Nepal especially in the Terai region (bordering India), while others said that they have no information about such commercialization. One respondent said, *“Syringes are being collected by the scrap handlers to be recycled for plastic. There might be potential for illegal repacking because the syringes collected are not damaged and it can be reused (repacked) after simple cleaning. ……… the plastic of syringe has high value compared with other plastics used in health care”* (P-8).

All respondents thought that the syringes and needles are discarded in safety boxes immediately after use, but a few of them had a reservation that the practice is limited to only a few health care facilities. A representative statement was*, “Not in every hospital and health centre the syringes and needle are appropriately disposed”* (P-7).

Most respondents believed that the used syringes are disposed of properly in Nepal, while others believed that the disposal is not satisfactory. Among those who believed that the disposal of used syringes is satisfactory, a few respondents believed that the safe disposal of used syringes is limited to central level hospitals and government primary health facilities only. Almost all policymakers thought that health care institutions have a waste management plan, but more than half of them were of the opinion that such plans are limited to only a few tertiary care hospitals in the country’s capital. One of the policymakers shared that the government of Nepal has been planning to extend the waste management system (implemented only in a few government tertiary care hospitals) to zonal hospitals in a few years. Only one policymaker said that health care services do not have a waste management plan. Lack of manpower, training, and monitoring systems, along with negligence and budget constraints, were thought to be hindering factors for such plans.

The respondents unanimously agreed that injection safety is a problem in Nepal. Most policymakers shared that negligence, lack of awareness about safe injection practices and risks of unsafe injection practices were the main factors responsible for unsafe injection practices. Similarly, poverty, misconceptions about injection efficacy, underestimation of the risks of unsafe injection practices and advertisement of injections in dispensaries were stated to be important factors contributing to unsafe practices. Lack of injection practice skills among HCWs was also mentioned by a few policymakers. One participant stated, *“Lack of… knowledge and skills* [for injection practice] *among HCWs especially among newly recruited employees cause unsafe injection practices”* (P-3). Similarly, another participant shared, “*Allowing patients and visitors to transfer used syringes e.g. blood samples to the laboratory also contributes to unsafe practices”* (P-11)*.* In Nepal, health institutions (even tertiary care hospitals) do not have a proper system to transfer blood samples, especially those of admitted patients. Nursing staff generally perform phlebotomy and direct the patient’s attendant to carry the sample to the respective laboratory for diagnostic tests. These samples are sometime carried in a disposable syringe with a capped needle.

## Discussion

The study explores the perception of policymakers about injection practice in Nepal and initiatives taken by the government for improving injection practice.

### Types of injection providers

For analysis, the injection providers were classified into formal, informal and quack sectors. The formal sector includes doctors, nurses and other health care workers who are qualified, trained and has legal rights to administer injections. The informal sector includes traditional healers, medical dispensers (pharmaceutical personnel) among others who are neither trained nor have legal rights to administer injections. They are trained for other health care services, e.g. medical dispensers are trained for pharmacy practice but not for injection administration. The informal sector has strong professional associations and may have influence at the higher levels of government, [9] while the quacks comprise of individuals who are not formally identified by the state. The quacks are either self-taught or learn the injection procedure by observing it being carried out by another person. Almost all policymakers shared that formal and informal health providers both administer injections. Two-thirds of the policymakers believed that quacks also administer injections to people in Nepal. Administration of injections by unqualified personnel and quacks was also reported a few years back in central Nepal [13]. Almost two-thirds of the policymakers believed that injections were overused in Nepal. Both overuse and administration of injections by unqualified personnel make injections unsafe [10].

### Initiatives to reduce injection overuse

For safe injection practice, unnecessary injections should be avoided. The National Drug Policy (1995) of Nepal aims to promote rational use of medicine through regular training and implementation of STS. There is a separate subsection regarding “Prudent use of antibiotics” in the policy (added by amendment in 2001), but there is still no such section for injection use [22]. Almost half of the policymakers, especially in MoHP, were of the opinion that the National Drug policy (NDP) of Nepal discourages injection overuse, while others, including DDA authorities, mentioned that the NDP does not directly discourage injection overuse. This may be because national drug policy discussions could have been less extensive among MoHP personnel who may be more concerned about the overall National Health Policy.

Effective implementation of the essential medicine policy and the STS/standard treatment guideline (STG) emphasizing the rational use of oral formulations rather than injectable formulations also helps to check overuse [6]. Both the policy and schedule should be implemented properly for better outcomes. Minimizing the number of injections in EDL and STS was reported as an initiative by the policymakers. As the EDL and STS have been implemented in government health care facilities, the decrease might have been limited to those health care facilities only. Furthermore, the EDL was revised after 9 years in 2011 and the STS has not been revised during the last 15 years [23]. Since 2010, the government of Nepal has implemented a policy of making basic health services accessible to the people and as a part of this policy [3], a few essential medicines are provided free of cost from government health care facilities (up to district level hospitals). As this list contains a reduced number of injections, policymakers stated that the overuse of injections has been discouraged. However, the authors are of the opinion that only decreasing the number of injections in the EDL or decreasing the supply may not be sufficient to decrease injection overuse, because even if injections are not available in government health care facilities, injection providers may provide injections in their private clinics [15].

### Initiatives for safe injection practice

Use of auto disable (AD) syringes for immunization significantly decreases the disease burden due to unsafe injection practices [12]. Using single-use disposal syringes (especially AD syringes for immunization) and matching the supply of injection equipment and diluents with the delivery of injectable medicine and vaccines, as shared by most policymakers, prevents reuse of injection equipment and are important for safe injection practices. The matching of injection equipment (including syringes) ensures that the equipment is available continuously and in sufficient quantity, which is a key determinant to ensure safe injection practices [24]. Shortages of these devices lead to their reuse [6,24,25] and promote unsafe injection practices.

Increased demand for safe injections and for oral substitutes of injections could also be promoted through educating people about the hazards of unsafe injections [26]. Communicating the risk of HIV infection associated with injections to the general public and to health care workers (HCWs), as shared by most policymakers, is also important to ensure safe injection practices and reduce unnecessary injections. However, the educational interventions thus planned should not adversely affect the demand for essential injections such as vaccines and contraceptives.

#### Quality monitoring of injection equipment

Almost two-thirds of the policymakers reported that there was no legally authorized body to check and control the quality of injection equipment in Nepal, while other policymakers were either unaware of or lacked clarity about the authority. In Nepal, most injection devices are imported, with a majority coming from India [15] where illegal reuse and repacking of used syringes is common [27,28]. Almost half the policymakers acknowledged that illegal commercialization of used disposable syringes exist in Nepal. Hence, there may be reason to suspect the quality of the syringes available in the national market. The Guidelines of Regulation of Health Related Products, as mentioned by a few high-level authorities of MoHP, is expected to strengthen the quality monitoring of health related products, including injection equipment. Implementation of this guideline, which is still at the drafting stage, is the need of the hour.

#### Disposal of used injection equipment

Use of safety boxes is important for the safety of injection providers. Studies have shown that a significant proportion (5-28%) of NSIs are due to unsafe sharp waste collection procedures [29,30] and use of safety boxes for collection of sharp waste reduces the risk [31]. Hence, a supply of safety boxes could be considered a positive initiative towards safe injection practice.

In Nepal, certain policies and strategies have been framed to address the management of general waste and hospital waste. The most important of these were the Health Care Waste Management Guidelines (2008/9) and the Solid Waste Management Act (2011) [32]. The Health Care Waste Management Guidelines (2008/9) were regarded as an institutional waste management policy by almost all policymakers, but the policy is not implemented satisfactorily [32]. The guidelines have categorized health care wastes and emphasized waste minimization, segregation and proper disposal. They have also differentiated the responsibilities of waste producers (health institutions), local authorities and national-level hospitals [33]. The Solid Waste Management Act (2011) deals with the management of all kinds of waste, including biomedical waste. According to the act, the management of solid waste (including biomedical waste) is the responsibility (legally and financially) of the health care facilities [34]. The implementation of both the guideline and the act are poor [32]. The National Heath Policy, 1991 does not address medical waste management, so the issue of medical waste management and environmental health should be addressed in an integrated manner [16] because proper sharp waste management is crucial for safe injection practice [10].

### Recommendations

Use of sterile injection equipment (syringes) for injections, ensuring availability of safety boxes, formulating and implementing guidelines and plans for disposal of used syringes, and educating the general public and HCWs about the association between injections and infections through HIV/AIDS prevention programmes (as stated by the policymakers in this study) could be initiatives to promote safe injection practices. The use of AD syringes and safety boxes, which at present is limited to child immunization services and government health institutions, should be broadened. Lack of injection-related policies and the improper implementation of guidelines and plans for health care waste management may be considered as grey areas for safe injection practice. Hence, policies and guidelines to monitor the quality of injection equipment (including syringes), restricting injection practice by unqualified health care professionals and quacks, and safe and environment-friendly disposal of injection equipment is recommended. Furthermore, effective implementation and regular updating of EDL and STG is required to reduce injection overuse.

### Limitations

To the best of our knowledge this is the first study in Nepal conducted with the objective of obtaining the perception of important policymakers about injection practices in the country. The sample size was small and though heads of important departments dealing with injection safety in Nepal were included there is a possibility that the findings may not be fully representative of the opinion of health policy makers and policy makers not included in the study may have a different perspective. The result may however, provide a platform for further studies. Further studies that also include stakeholders from the community may provide a clearer picture about the injection practice in Nepal. Repeat interviews with policymakers could not be done due to time constraints. Instances of recall bias may have occurred among the participants.

## Conclusions

Safe injection practice is a very important step toward ensuring safe and effective healthcare. This study indicates that the government of Nepal has attempted to make injection practices safer, but these attempts seem to be limited to immunization services and to government health care facilities. The study also found a diversity of views among policymakers about certain aspects of safe injection practice in Nepal. Proper coordination among policymakers and standardization of policies and procedures related to injection practice are important steps towards making injection practice safer. There is a need of further community-based surveys to expand the results of this study and to obtain a clearer picture of injection practices in Nepal. This study will serve as a platform for such surveys.

## Competing interest

All the authors declare that they have no competing interests.

## Authors’ contributions

SG, DSR, PRS and VVKC conceived and designed the study. SG collected data. SG, MM and VKKC synthesized, analyzed, and drafted the manuscript. DSR and PRS helped to interpret findings and review drafts of the manuscript. The final manuscript has been read and approved by all the authors.

## Pre-publication history

The pre-publication history for this paper can be accessed here:

http://www.biomedcentral.com/1472-698X/14/21/prepub
